# Impact of short-term heat treatment on the structure and functional properties of commercial furcellaran compared to commercial carrageenans

**DOI:** 10.1016/j.heliyon.2021.e06640

**Published:** 2021-04-08

**Authors:** Kairit Eha, Tõnis Pehk, Ivo Heinmaa, Aleksei Kaleda, Katrin Laos

**Affiliations:** a Department of Chemistry and Biotechnology, Tallinn University of Technology (TTU), Akadeemia tee 15, 12618, Tallinn, Estonia; b National Institute of Chemical Physics and Biophysics, Akadeemia tee 23, 12618, Tallinn, Estonia; c Centre of Food and Fermentation Technologies, Akadeemia tee 15A, 12618, Tallinn, Estonia

**Keywords:** Carrageenan, NMR, Molecular weight, Rheology

## Abstract

In the production of biopolymers, the processing operations (e.g. extraction and drying) involve some degradation of the polysaccharide-causing structural and functional changes in final products. In this study, short-term heat treatment (75–115 °C, 15 min) influence on commercial carrageenans' — furcellaran, κ-carrageenan, ι-carrageenan and a κ/λ-carrageenan — structure, molecular weight and gel rheology was studied. Compared with other carrageenans, commercial furcellaran that had undergone multiple heatings at high temperatures during production was found to be susceptible to polymer degradation. Heat caused the desulphation and degradation of furcellaran galactans and the molecular weight was significantly decreased, causing a drop in viscosity and gel hardness. The loss of the network cross-linking of furcellaran gels was confirmed by scanning electron microscopy. Carrageenan gel storage modulus values decreased with the increase in the temperature of the treatment. The greatest decrease in storage modulus values occurred with κ/λ-carrageenan gels, followed by ι-carrageenan > furcellaran > κ-carrageenan.

## 1 Introduction

Carrageenans are linear sulphated polysaccharides obtained from red algae. Their basic structural units are disaccharides consisting of alternating β-1,3- and α-1,4-linked galactose residues. The differences in the basic structure are due to the occurrence of 3,6-anhydrogalactose and sulphate groups' position and number in linked galactose residues [1]. The most important carrageenans are the κ− (KC), ι− (IC) and λ− (LC) types, which are differentiated by the occurrence of one, two or three sulphate ester groups per repeating disaccharide unit, respectively [2]. Commercially these types are often mixed or they are natural hybrid molecules consisting of different types of carrageenans [3].

Furcellaran (Fur) is similar in structure to KC, but the main difference is a lower sulphation level [4]. Structural complexity occurs when the hydroxy groups in D-galactose are replaced by sulphate, methyl and pyruvate groups [5, 6].

Carrageenans are used as gelling, thickening and stabilising components for various industrial purposes, such as pharmaceuticals, cosmetics and foods [7]. Different types of carrageenans give a wide spectrum of textures. Fur, KC and IC are gel–forming carrageenans, whereas LC is used only as a thickening agent. Carrageenan gelation is a two-step process: helical formation upon cooling and a further helical cation-specific aggregation [8]. Sulphate arrangements greatly influence the functional properties of carrageenans. Sulphation at the C2 position of (1→3) linked β-D-galactopyranose residues generally reduces the gel-forming ability of carrageenans by avoiding the formation of a helical structure [9]. The gel strength of different types of carrageenans depends on the presence and content of 3,6-anhydro-D-galactose residues [10]. Fur forms strong gels, similar to KC. The latter forms rigid and brittle gels, while IC forms soft and elastic gels [7].

The specific details of commercial carrageenan production processes are classified, but in general the production technology is rather similar. The extraction of carrageenans from weeds is carried out with hot water or an alkaline solution [11, 12, 13]. The alkaline treatment releases sulphate ester groups, causes the formation of 3,6-anhydro-D-galactose units and improves gel strength [14]. Several methods have been used to recover carrageenans from solutions. Higher quality products are obtained by the precipitation of carrageenans from solutions by alcohols [15]. However, as the alcohol precipitation method cost is higher than the direct drying method cost, spray drying or steam-heated rolls have been used extensively for concentrated carrageenan filtrates [16]. The elevated and uneven conditions during direct drying may initiate the degradation of polysaccharide macromolecules and consequently can affect the gels' rheological and mechanical properties. Robal et al. [17] have studied carrageenans' thermal stability in dry and sol states. They found that degradation during heat treatment in the dry state depends on the galactan's sulphur content and decomposition begins at lower temperatures for more sulphated preparations. They also found that thermal degradation is more intensive in the presence of divalent cations and decreases in the presence of methoxy groups. For drum-dried Fur, intense polymeric chain destruction was reported to begin at temperatures above 115 °C and the product survived brief heat treatment (15 min at 130 °C), after which a remarkable gel strength decrease was observed [18]. The same Fur powders dried at higher temperatures showed higher heat release [19], resulting in colour change and spontaneous combustion.

However, there have been no studies on the structural and functional changes in this drum-dried Fur after thermal processing at elevated temperatures. A better understanding of Fur properties will ultimately lead to better process control to obtain the best possible final product quality and increased consumer acceptance. The study's objective was to investigate short-term heat treatment effect on the structure and functional properties of drum-dried furcellaran compared with other carrageenans.

## 2 Materials and methods

### 2.1 Materials

Commercial furcellaran was extracted from *Furcellaria lumbricalis* (Gigartinales) (AS EstAgar, Kärla, Estonia). The furcellaran production process includes drying on rollers in the final step. Commercial κ-carrageenan, ι-carrageenan and a mixture of κ- and λ-carrageenan (kappa and lesser amounts of lambda carrageenan) preparations were purchased from Sigma (product codes 22048, C1138 and C1013, respectively).

### 2.2 Heat treatment

The heat treatment of the samples was performed as described by Friedenthal et al. [18] with a Halogen Moisture Analyzer HR 73 (Mettler Toledo, Switzerland) at 75–115 °C, with the treatment time being 15 min.

### 2.3 Chemical analysis

Monosaccharide contents were obtained by the hydrolysis of the polysaccharides in 2 M H_2_SO_4_ at 110 °C for 60 min, followed by neutralisation with 1 M NaOH; the monosaccharides were quantified by high-performance anion-exchange chromatography (HPAEC-PAD) [20], using the Shimadzu Prominence HPLC system (Shimadzu, Japan), equipped with an Antec II Decade electrochemical detector (ANTEC Leyden, The Netherlands). The analysis was carried out on a Dionex CarboPac MA-1 column, thermostated at 35 °C. Elution was performed using 450 mM NaOH at a flow rate of 0.4 ml min^−1^, and the sample injection size was 10 μl.

The sulphate content was quantified using the BaCl_2_-gelatin turbidity method [21] after hydrolysing the samples in 1 M HCl at 115 **°**C for 5 h.

The pH of 1% (w/v) polysaccharide sols was determined using a Mettler Toledo SevenEasy pH meter.

### 2.4 NMR spectroscopy

^13^C NMR spectra were recorded on a Bruker AVANCE III spectrometer operating at 800 MHz. The spectra of 2.5% polysaccharide solutions in D_2_O (w/w) were obtained at 60 °C, and 30000 transients were collected with a 1 s inter-pulse delay. The chemical shifts were calculated with reference to the C-6 signal from the galactose subunit, having a constant value of 61.3 ppm for these carrageenans [22].

^13^C CP-MAS NMR spectra were recorded on a Bruker AVANCE-II spectrometer at 14.1 T magnetic field using cross polarisation, a proton decoupling pulse sequence and a home-made double resonance probe with magic-angle-spinning for 4 × 25mm Si_3_N_4_ rotors. The sample spinning speed was 12.5 kHz, the ramped polarisation transfer pulse duration was 1 ms, and the relaxation delay was 5 s.

### 2.5 Size exclusion chromatography

The molecular weight (Mw) determination was carried out in polysaccharides through size exclusion chromatography (SEC) analysis following the method of Saluri et al. [23], using a Shimadzu LC-30AD liquid chromatograph equipped with a RID-10A refractive index detector, a Shimadzu CTO-20AC column oven, an OHpak SB-G guard column, and two Shodex OHpak SB-806MHQ columns in series. Elution was conducted using a 0.1 M NaNO_3_ solution as the mobile phase flow rate was set at 0.8 mL min^−1^. The column oven temperature was set at 60 °C. To estimate the peak-average Mw, a calibration curve was obtained from 12 pullulan standards.

### 2.6 Lightness

The dry Fur sample lightness was evaluated with a CM-700d spectrophotometer (Konica Minolta, Japan), CIE D65/11 mm/2°.

### 2.7 Rheological characteristics of carrageenan sols and gels

#### 2.7.1 Preparation of sols and gels

All hydrocolloid solutions were prepared by dissolving carrageenan powders in distilled water at 75 °C with a magnetic stirrer. After the complete solubilisation of the polysaccharides, the sols were used for viscosity and temperature sweep tests or poured into moulds (20 × 20 × 20 mm), cooled to +5 °C and stored at that temperature overnight for other rheological measurements.

The polysaccharide concentrations were 2.5% (w/v) in all cases, except for the temperature sweep test, where the polysaccharide concentration was 1.5% (w/v).

#### 2.7.2 Viscosity

The flow properties of 2.5% (w/v) polysaccharide sols at 75 °C were measured with a RheolabQC rotating viscometer (Anton Paar, Germany), fitted with a concentric cylinder measuring set CC27 system, with a temperature-controlled water bath. The sample volume was 20 ml and the apparent viscosity η (Pa∗s) was measured as a function of shear rate γ̇ (10–50 s^−1^) for 40 s.

#### 2.7.3 Hardness

The hardness (first peak height) was determined using a TA-XT2i texture analyser (Stable Micro Systems, Surrey, England). Gel samples were removed from the fridge and moulds (20 × 20 × 20 mm) and allowed to equilibrate to room temperature for 2 h before testing. A stainless steel cylindrical probe (25 mm in diameter) was used to compress the samples to 50% of their original height at a constant speed of 1 mm s^−1^.

#### 2.7.4 Rheology

A dynamic rheological measurement was performed with an MCR 301 rheometer (AntonPaar GmbH, Germany), using a serrated parallel plate of 50 mm diameter (profile depth: 0.5 mm) to minimise slippage at the gel-geometry interfaces. The gap between the plates was kept at 1 mm. All measurements were performed in duplicate, and data points were recorded at steady state.

The storage modulus (G′) and loss modulus (G″) of the gels at room temperature were determined by the modified method proposed by Chen et al. [24]. The study consisted of the following steps: time sweep (2 min) at 10 Hz and 0.1% strain (within the viscoelastic region), followed by a frequency sweep from 0.01 to 100 Hz at 0.1% strain. After another time sweep (2 min) at 0.1% strain 10 Hz measurement, an amplitude sweep at 0.01–100% strain 10 Hz, and a final time sweep (2 min) at 0.1% strain 10 Hz were recorded.

For temperature sweeps, the polysaccharide solutions were poured on a pre-heated (90 °C) Peltier plate and its borders were coated with low-viscosity silicon oil to prevent water loss. After 5 min, the samples were cooled down to 25 °C at a rate of 1 °C min^−1^ at 0.1% strain and 10 Hz frequency to follow the gelation process. The sample melting process was assessed during heating from 25 to 90 °C, at a constant rate of 1 °C min^−1^ and under the same strain and frequency conditions.

### 2.8 Scanning electron microscopy

2.5% (w/v) polysaccharide gels were frozen in liquid nitrogen and freeze-dried under vacuum at -60 °C. For scanning electron microscopy (SEM), samples were mounted on aluminium sample holders and were transferred to a SEM unit equipped with an EPSE detector (EVO LS15, Carl Zeiss, Milan, Italy), which was at ambient temperature and filled with water vapour at 70 Pa pressure.

## 3 Results and discussion

### 3.1 Characterisation of carrageenans

#### 3.1.1 Chemical analysis

Table 1 shows the commercial carrageenan powder chemical compositions. The sugar analysis indicated that the most important sugar in all of the samples was galactose, followed by 3,6-anhydrogalactose. A high sucrose content was found in the KC samples. Such additives as sucrose and glucose are often used to improve commercial carrageenan preparations' functional properties (e.g. solubility, viscosity and gel strength). Small glucose amounts were found in IC and KC/LC samples, likely derived from floridean starch and 6-O-methylgalactose in the Fur samples. The highest ester sulphate content was found in the IC (36.1 % w/w, approximately two groups per repeating disaccharide unit) and the lowest in the KC (15.7 % w/w).

**Table 1:** Monomeric composition (%, w/w dry weight) of commercial carrageenans.

Sample	Glucose	Sucrose	Galactose	3,6-anhydro-galactose	6-0- methyl galactose	SO_4_
Fur	-	-	39.2 ± 0.5	29.4 ± 0.6	2.1 ± 0.2	16.2 ± 0.4
KC	-	26.3 ± 0.5	26.3 ± 0.4	25.4 ± 0.5	-	15.7 ± 0.3
KC/LC	1.2 ± 0.1	-	27.6 ± 0.5	26.0 ± 0.4	-	22.9 ± 0.3
IC	1.4 ± 0.1	-	28.5 ± 0.4	22.8 ± 0.4	-	36.1 ± 0.4

The KC sample sulphur content was lower than expected, but this can be attributed to the presence of sucrose additive. Excluding the additive, the KC sulphur content was approximately one group per repeating disaccharide unit, a value that is in agreement with the data reported elsewhere [25, 26].

#### 3.1.2 ^13^C-NMR

**Figure 1:**
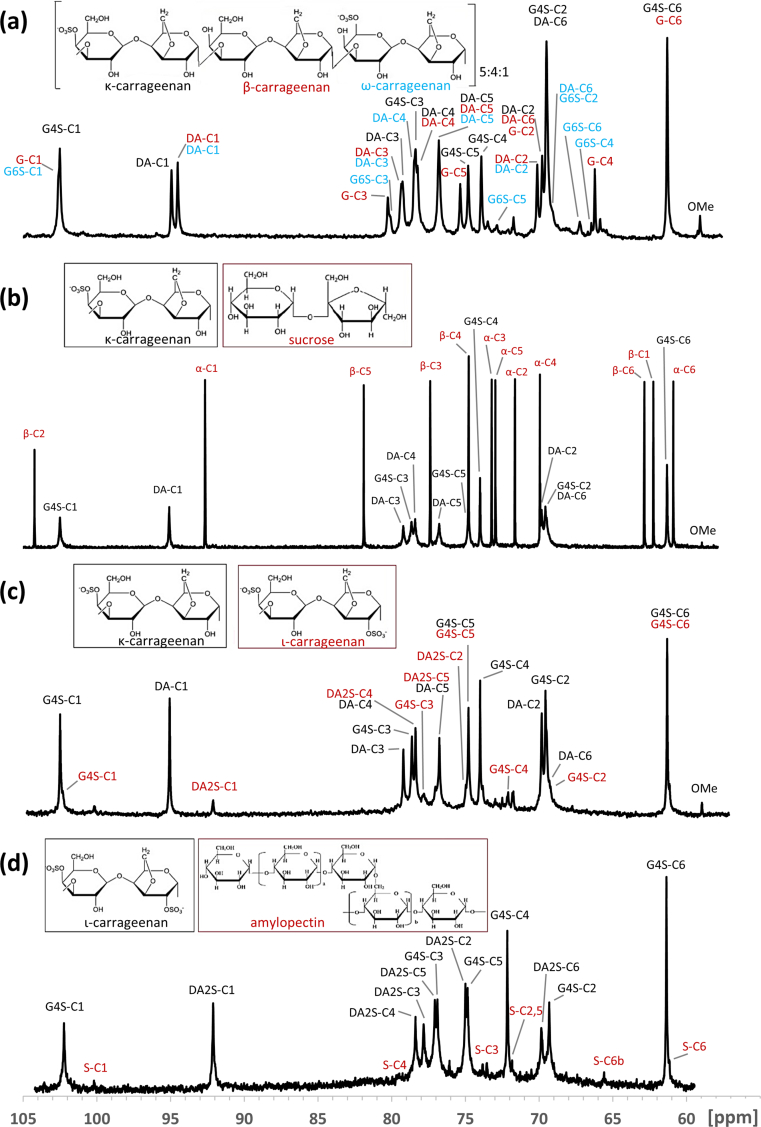
^13^C-NMR spectra of carrageenans. ^13^C-NMR spectra of carrageenans. (a) furcellaran, (b) κ−carrageenan, (c) κ/λ−carrageenan, (d) ι−carrageenan.

^13^C-NMR spectra of the analysed samples are shown in Figure 1. The signal assignments (Table 2) are based on the carrageenan structure chemical shifts [26].

**Table 2:** ^13^C-NMR chemical shift assignments (ppm) of commercial carrageenans.

Sample	Residue	Unit	Chemical shifts (ppm)					
			C1	C2	C3	C4	C5	C6
Fur	κ−carrageenan	G4S DA	102.5 95.0	69.5 69.8	78.5 79.2	74.0 78.3	74.8 76.8	61.3 69.5
	β−carrageenan	G DA	102.6 94.5	69.7 70.1	80.3 79.3	66.4 78.3	75.4 76.8	61.3 69.7
	ω−carrageenan	G6S DA	102.6 94.6	69.4 70.1	80.2 79.3	66.2 78.5	72.9 76.8	67.2 69.4
KC	κ−carrageenan	G4S DA	102.5 95.0	69.5 69.8	78.7 79.2	74.0 78.3	74.8 76.8	61.3 69.5
	sucrose	α−D-glucopyranose β-D-fructopyranose	92.9 62.2	71.9 104.5	73.4 77.3	70.0 74.8	73.2 82.2	61.0 63.2
KC/LC	κ−carrageenan	G4S DA	102.5 95.3	69.6 69.9	78.9 79.2	74.1 78.3	74.8 76.8	61.3 69.5
	ι−carrageenan	G4S DA2S	102.2 92.1	69.3 75.0	76.8 77.8	72.1 78.3	74.8 77.0	61.3 69.8
IC	ι−carrageenan	G4S DA2S	102.2 92.1	69.3 75.0	76.8 77.8	72.1 78.3	74.8 77.0	61.3 69.8
	starch	amylopectin	100.2	72.2	73.5	79.2	71.8	61.1

The commercial Fur main components were (1→3) linked β-D-galactopyranose, (1→4) linked 3,6-anhydro-α-D-galactopyranose, (1→3) linked β-D-galactopyranose 4-sulphate and (1→3) β-D-galactopyranose 6-sulphate, indicating that Fur is a hybrid of κ−, β− and ω−carrageenan, which has been reported elsewhere [27, 28, 29], with an approximate ratio of 5:4:1, respectively. Also, 3-linked 6-O-methyl-D-galactose residues were found, as these residues give specific signals for OMe at 59.0, for the substituted C-6 at 71.8, and for the neighbouring C-5 at 73.3 ppm in ^13^C-NMR [30]. Additionally, several peaks between 10-35 ppm were registered (data not shown), indicating the presence of vegetable fats [31]. The fats could have got into the furcellaran during the production process when oil was added to the drums during the drum-drying process.

Commercial KC was found to be a blend of KC (the signals due to (1→4) linked 3,6-anhydro-α-D-galactopyranose and (1→3) linked β-D-galactopyranose 4-sulphate) and sucrose, with an approx. 1:1 M ratio. Also, 3-linked 6-O-methyl-D-galactose residues were found.

The spectrum of KC/LC shows the prevalence of KC but no signals of LC were detected. However, the signals of minor components indicate the presence of IC. Being common to KC, 6-O-methyl-D-galactose residues were again found.

The main commercial IC signals were due to 3-linked β-D-galactopyranose 4-sulphate and 4-linked α-D-galactopyranose 2,6-disulphate, corresponding to those of IC. Additionally, peaks corresponding to amylopectin were registered, indicating the presence of starch in the product, with branch-point residue C-6_b_ detectable at 65.5 ppm [32].

### 3.2 Effect of heat treatment on the structural properties of carrageenans

#### 3.2.1 ^13^C CP-MAS NMR

**Figure 2:**
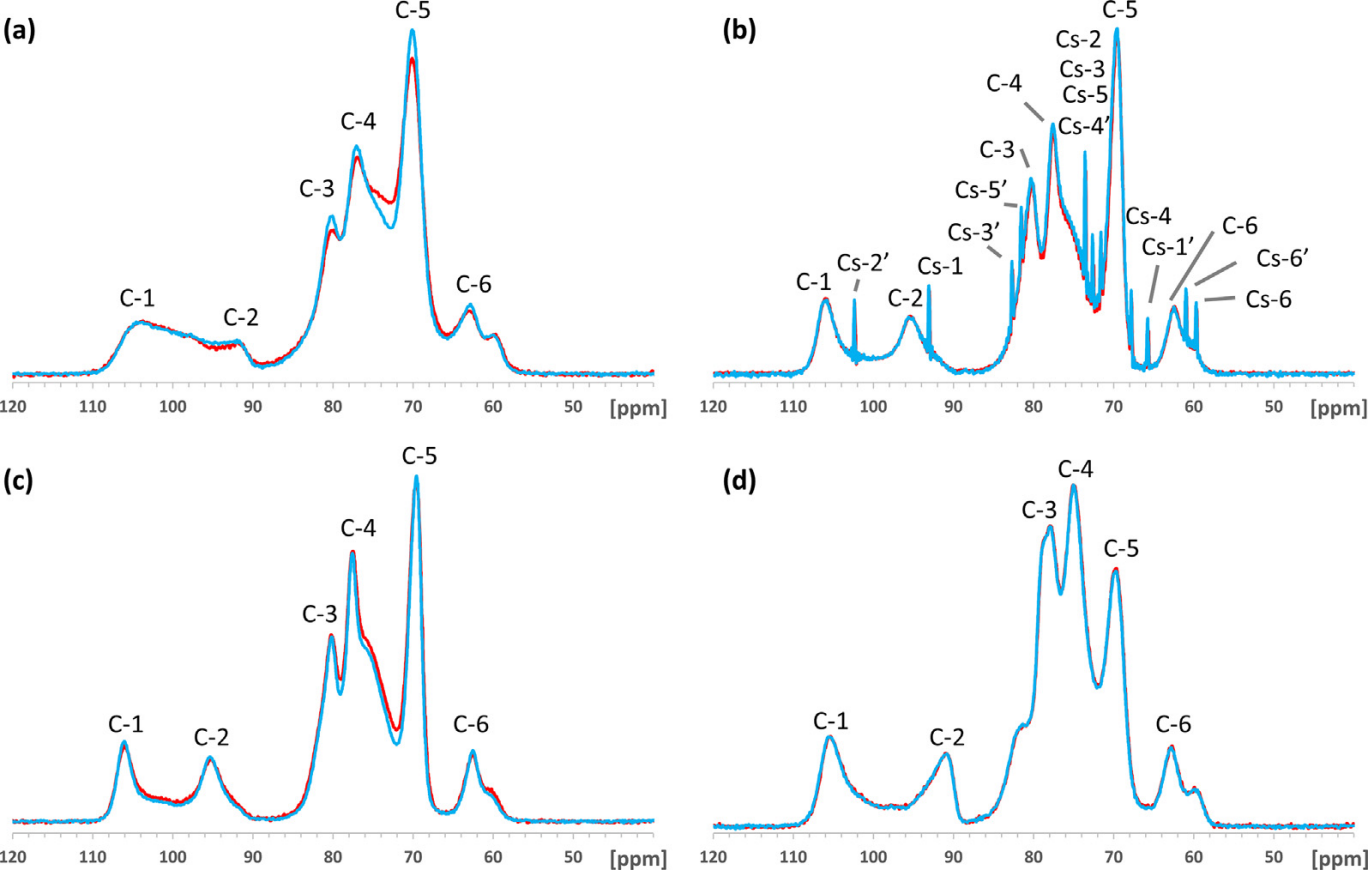
Comparison of the carrageenan spectra before (blue line) and after (red line) heat treatment. (a) furcellaran, (b) κ−carrageenan, (c) κ/λ−carrageenan, (d) ι−carrageenan. Chemical shifts from C-1–C-6 correspond to polysaccharide; chemical shifts from C_s_-1–C_s_-6 and C_s_-1′–C_s_-6′ correspond to sucrose.

The ^13^C CP-MAS NMR spectra of carrageenans show six signals (Figure 2) representing the galactopyranose and the anhydrogalactopyranose residues. The signal assignments (Table 3) are based on the carrageenan structure chemical shifts [33]. As seen with ^13^C-NMR, the KC spectra showed an additional 12 peaks corresponding to sucrose chemical shifts [34, 35] (Table 3). Excluding the sucrose signals, the KC, KC/LC and Fur chemical shifts were very similar. The KC/LC spectrum showed chemical shifts corresponding only to KC; chemical shifts corresponding to LC [33] were not seen. Comparing the Fur with the KC spectrum, the resonance of the C-4 signal was of lower intensity, to the advantage of the C-5 signal, which was increased. As Fur is partially desulphated KC, due to the presence of the unsubstituted hydroxyl on 4G, this carbon resonates up field [36].

**Table 3:** ^13^C CP-MAS NMR chemical shift assignments (ppm) of commercial carrageenans and their additives.

Sample	Residue	Peak	Chemical shift
Fur KC KC/LC	1G	C-1	104.5
	1A	C-2	91.9
	3A, 3G, 4A	C-3	80.1
	5A, 5G, 4G	C-4	77.0
	2A, 2G, 6A	C-5	70.1
	6G	C-6	62.8
KC KC/LC	1G	C-1	105.9
	1A	C-2	95.5
	3A, 3G, 4A	C-3	80.2
	5A, 5G, 4G	C-4	77.6
	2A, 2G, 6A	C-5	69.6
	6G	C-6	62.5
IC	1G	C-1	105.7
	1A	C-2	90.8
	3A, 3G, 4A, 5A	C-3	77.9
	2A, 5G	C-4	75.0
	4G, 2G, 6A	C-5	69.7
	6G	C-6	62.9
Sucrose	α-D-glucopyranose	Cs-1	93.0
		Cs-2	73.6
		Cs-3	72.6
		Cs-4	67.8
		Cs-5	73.6
		Cs-6	59.7
	β-D-fructopyranose	Cs-1′	65.8
		Cs-2′	102.4
		Cs-3′	82.7
		Cs-4′	71.6
		Cs-5′	81.5
		Cs-6′	61.0

G – galactopyranose residues.

A – anhydrogalactopyranose residues.

The relative changes in intensity of the peaks C-3, C-4 and C-5 between the spectra of KC and IC are based on the NMR substitution rules applied to sulphation [37]. Indeed, IC can be considered KC with the hydroxyl linked to carbon 2A replaced by a sulphate group.

No heat treatment influence at 115 °C for 15 min on the carrageenans' structure was seen (Figure 2), except for Fur, where the intensity of the peaks from C-3 to C-6 decreased, indicating polysaccharide degradation and galactan desulphation.

**Figure 3:**
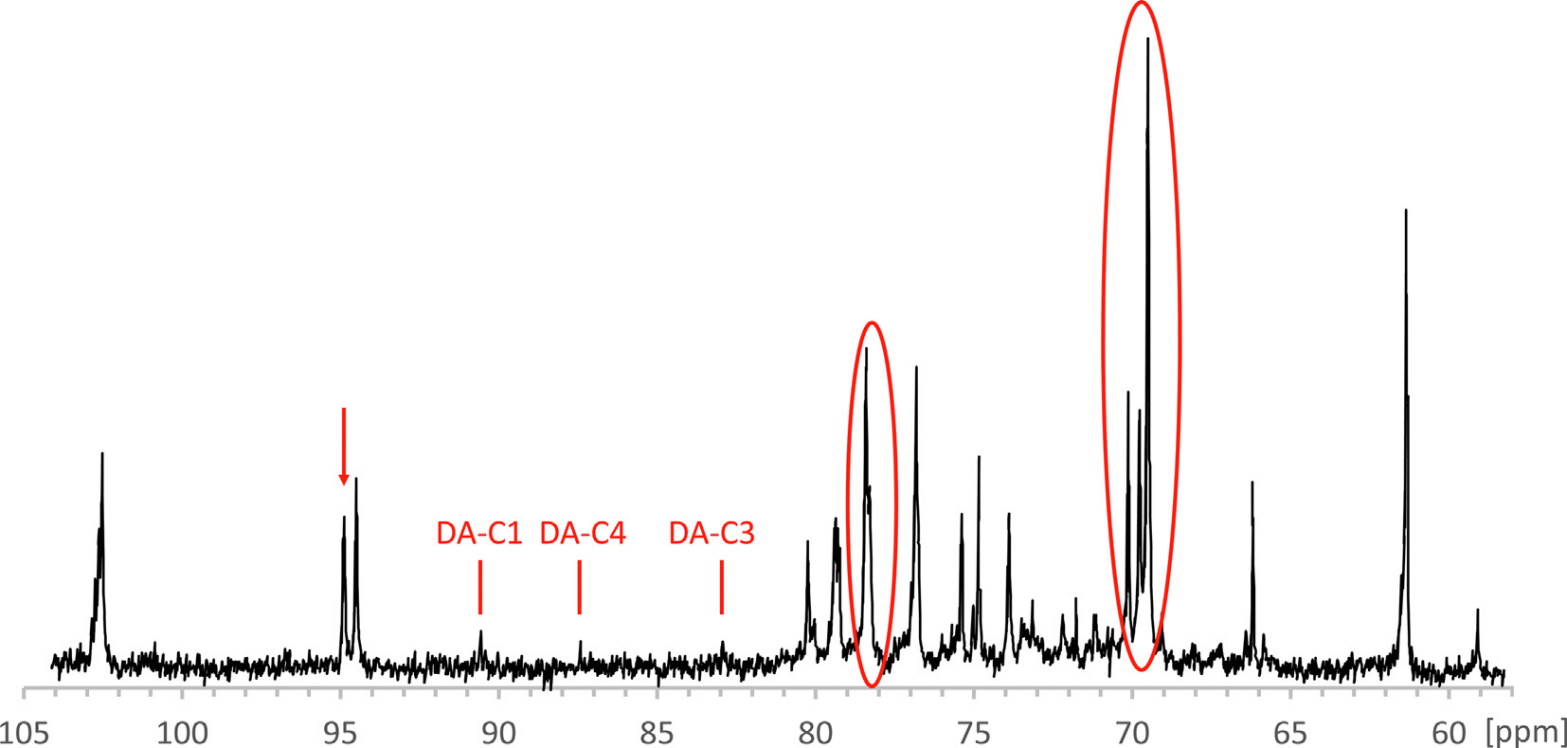
^13^C-NMR spectra of furcellaran dry-heated at 115 °C 15 min.

In order to further investigate the structural differences between non-treated and heat-treated carrageenans, the ^13^C-NMR was applied. Again, no short heat treatment influence on the carrageenans’ structure was seen (data not shown), except for Fur (Figure 3). Compared with non-treated Fur (Figure 1), three additional peaks, at 90.4, 87.2 and 82.8 ppm (DA-C1, DA-C4, and DA-C3, respectively), appeared, indicating the formation of oligosaccharides with decreasing terminal 3,6-anhydro-α-D-galactose residues [38]. Similarly to Robal et al.'s work [17], concurrent β-D-galactose and β-D-galactose-4-sulphate anomeric signals became segregated, with the latter showing slightly higher peaks in the spectrum. Also, a decrease in anomeric carbon of 3,6-anhydro-α-D-galactose residue at 95.0 ppm was observed, which can be explained by galactan desulphation [17].

#### 3.2.2 SEC

The average molecular weight of the carrageenans as a function of heat treatment temperature is shown in Table 4. Of the non-treated samples, KC had the highest average molecular weight (1460 kDa), followed by IC (1201 kDa) and KC/LC (1059 kDa). Fur had the lowest average molecular weight (252 kDa). These molecular weight differences can be explained by the different production processes. It is known that elevated temperatures and prolonged extraction and drying times can cause carrageenan degradation [17, 39]. The results indicated that the carrageenan degradation rate was accelerated at higher temperatures, as lower molecular weight carrageenans were obtained.

**Table 4:** Carrageenan molecular weight as a function of thermal treatment.

Sample	Molecular weight (Mw), kDa				
	Non-treated	75 °C	90 °C	105 °C	115 °C
Fur	252	194	179	91	22
KC	1460	1437	1420	1409	1389
KC/LC	1059	1047	1029	1028	1009
IC	1201	1183	1164	1133	1117

Fur showed more sensitivity to temperature than any other carrageenan. The molecular weight of Fur showed a huge drop at temperatures above 90 °C, and a 91% of average molar mass decrease was observed at 115 °C, while the other carrageenans showed a less than 7% of average molar mass decrease at the same temperature. It can be assumed that carrageenan degradation depends on the production process thermal history. As Fur is dried on drum-driers, it may bind more water compared with carrageenans [19], causing harsher degradation conditions.

#### 3.2.3 pH and lightness

**Figure 4:**
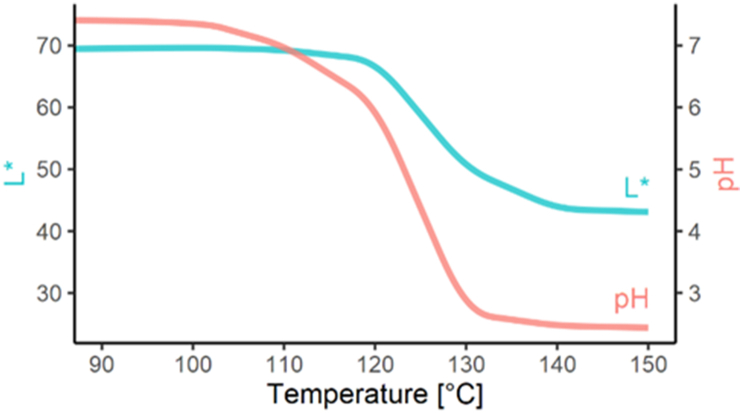
The dependence of furcellaran lightness (L∗) and 1% (w/v) furcellaran solution pH on the drying temperature.

The sample degradation at high temperatures was likely the result of the acid hydrolysis produced by the release of the sulphate groups, rendering them acidic. The formation of acidic degradation products was shown by the decrease in pH at higher treatment temperatures. The pH values of the non-treated KC, KC/LC and IC were 9.14 ± 0.01, 9.58 ± 0.01 and 9.45 ± 0.02, respectively. After heat treatment at 150 °C for 15 min, the carrageenans' pH slightly decreased to 9.05 ± 0.01, 9.51 ± 0.01 and 9.05 ± 0.01, respectively, and no colour change was observed. Compared with carrageenans, the non-treated furcellaran sample pH was lower, only 7.42 ± 0.02 (Figure 4), and had very low molecular weight, indicating that acidic degradation products had already formed during the Fur production process. During heat treatment, furcellaran pH showed a sharp decrease from 100 °C up to 130 °C, and started to plateau beyond this temperature, at a pH value of 2.4 ± 0.01. The degradation was accompanied by a colour change, to a deep brown/black tone, of the highly degraded Fur (Figure 4).

### 3.3 Heat treatment effect on carrageenan gels' rheological and microstructural properties

#### 3.3.1 Viscosity, hardness, gelling and melting temperatures

The non-treated and heat-treated (115 °C) 2.5% (w/v) carrageenan sols' viscosity at 75 °C is shown in Table 5. The highest viscosity was shown by KC, followed by IC and the KC/LC mixture. Fur had the lowest viscosity. These data correlate well with molecular weight (Pearson's r = 0.95). Higher molecular weight polymer coils occupy more solvent volume, resulting in an increase in solution viscosity.

**Table 5:** The viscosity and hardness of non-treated and heat-treated carrageenan sols and gels.

Sample	Viscosity, mPa∗s		Hardness, N	
	Non-treated	115 °C	Non-treated	115 °C
Fur	13 ± 1	1 ± 0	3 ± 0	0 ± 0
KC	102 ± 5	102 ± 4	12 ± 1	12 ± 1
KC/LC	47 ± 2	47 ± 3	21 ± 0	21 ± 1
IC	81 ± 4	81 ± 4	2 ± 0	2 ± 0

The heat treatment didn't affect the carrageenans' viscosity except for Fur, where a drop in viscosity was noticed. Similar results were observed when determining the 2.5% (w/v) carrageenans' gel hardness (Table 5). No changes in hardness values were observed for KC, IC or KC/LC gels after heat treatment at 115 °C. However, Fur formed thermally unstable brittle gels as the hardness decreased after the heat treatment. At 115 °C, Fur lost its gelling ability, apparently due to the loss of the minimum polysaccharide chain length required for the formation of ordered structures.

A rheological temperature sweep test was used for the determination of the gelling and melting temperatures of the sols/gels of 1.5% (w/v) carrageenans. All samples showed two distinct crossover points corresponding to the sol-gel transition temperature (T_sg_) during the cooling cycle and gel-sol transition temperature (T_gs_) during heating, confirming their thermo-reversible property. The T_sg_ and T_gs_ values are shown in Table 6. The KC/LC transition temperatures were much higher than for KC, probably due to the inclusion of IC. The highest transition temperatures were obtained with IC. It is apparent that both T_sg_ and T_gs_ shift to lower temperatures with a heat treatment temperature increase, indicating that heat treatment impedes coil-helix transition. Also, the thermal hysteresis between T_sg_ and T_gs_ decreased with heat treatment for all samples except for KC/LC. Fur showed the greatest shift in transition temperatures and in thermal hysteresis, probably due to the greater degradation of polysaccharide. A positive correlation between molecular weight and melting and gelling temperatures has been previously reported [15, 40].

**Table 6:** The gelling and melting temperatures of non-treated and heat-treated carrageenan sols and gels.

Sample	Sol-gel transition temperature (T_sg_), °C			Gel-sol transition temperature (T_gs_), °C		
	Non-treated	75 °C	95 °C	Non-treated	75 °C	95 °C
Fur	32 ± 0	28 ± 0	27 ± 2	53 ± 0	48 ± 0	45 ± 1
KC	33 ± 0	32 ± 0	32 ± 0	48 ± 0	44 ± 0	44 ± 0
KC/LC	45 ± 3	43 ± 1	42 ± 0	75 ± 0	73 ± 1	72 ± 1
IC	57 ± 0	55 ± 2	53 ± 1	60 ± 1	57 ± 1	56 ± 1

#### 3.3.2 Dynamic rheology

**Figure 5:**
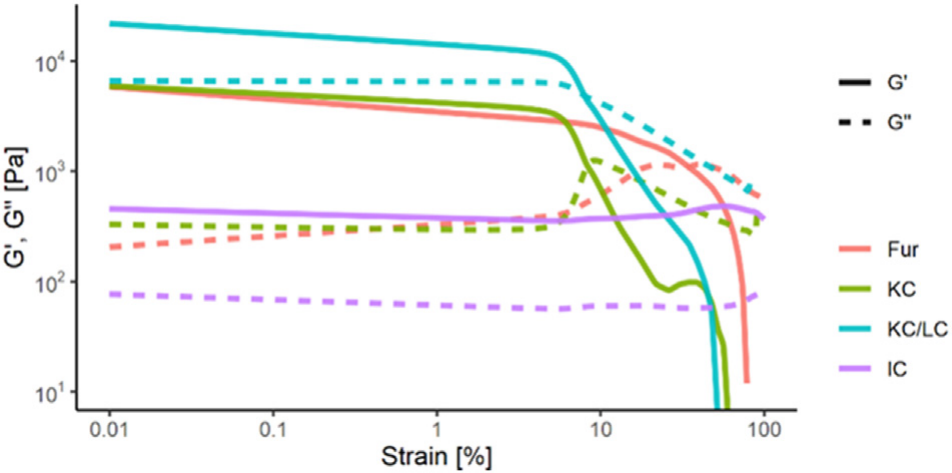
Storage modulus (G′) and loss modulus (G″) as a function of strain for 2.5% carrageenan gels.

Figure 5 shows storage modulus (G′) and loss modulus (G″) curves as a function of strain. The carrageenan gels present characteristic curves of well-structured systems with long linear viscoelastic regions reached up to 4% for KC and KC/LC gels and 10% for Fur gels, and decreasing trends over this threshold. The decrease can be explained by syneresis, which caused gel slippage between the plates of the rheometer.

No changes in G′ and G″ values were observed with IC. All G′ values were higher than G″, indicating a stable gel. However, an obvious difference appeared between the maximum G′ values of carrageenan gels due to their network strength differences. The highest G′ value was shown by KC/LC, followed by KC, Fur and IC. The gels with higher G’ values had more compact structures and were more stable at rest.

**Figure 6:**
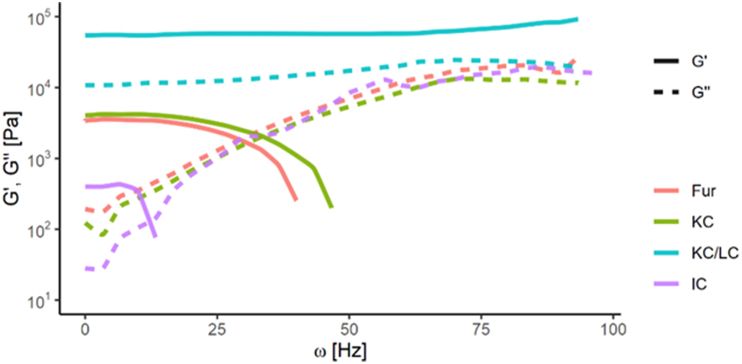
Storage modulus (G′) and loss modulus (G″) as a function of frequency for 2.5% carrageenan gels.

All studied polysaccharide gels showed a G′ vs. frequency dependence and can be classified into physically cross-linked network gels (Figure 6). The slightly rising G′ curve of the KC/LC gel was above the G″ curve over the entire applied frequency domain and the frequency of oscillations was little affected by the gel's viscoelastic properties. For other studied carrageenan gels, the crossover took place at lower frequencies (Fur at 30 Hz, KC at 33 Hz and IC at 13 Hz). This means that these samples act as reversible networks and can form gels at low frequencies and viscous sols at high frequencies.

**Figure 7:**
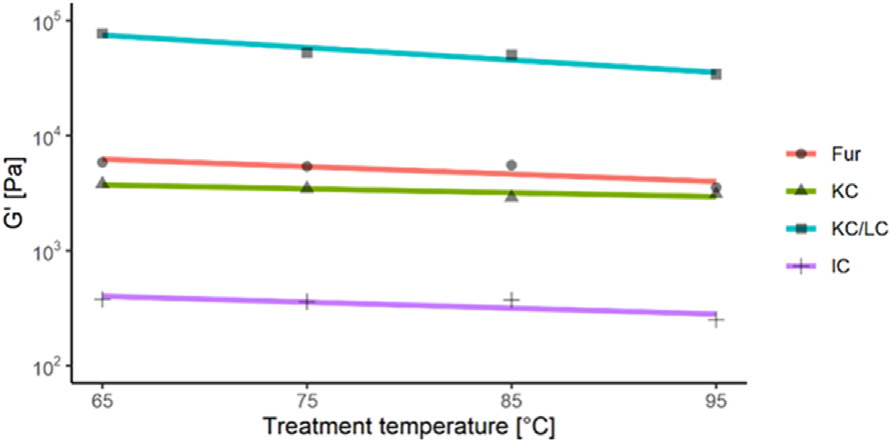
Storage modulus (G′) as a function of treatment temperature for carrageenan gels.

The G′ values of all studied carrageenan gels decreased with an increase in the heat treatment temperature (Figure 7). The greatest decrease in G’ values occurred with KC/LC gels, followed by IC > Fur > KC. As the hardness of the KC gels was higher than that of Fur, it can be concluded that Fur forms stronger but more brittle gels than KC does. IC forms soft and elastic gels, whereas KC/LC forms very strong and rigid gels.

#### 3.3.3 SEM

**Figure 8:**
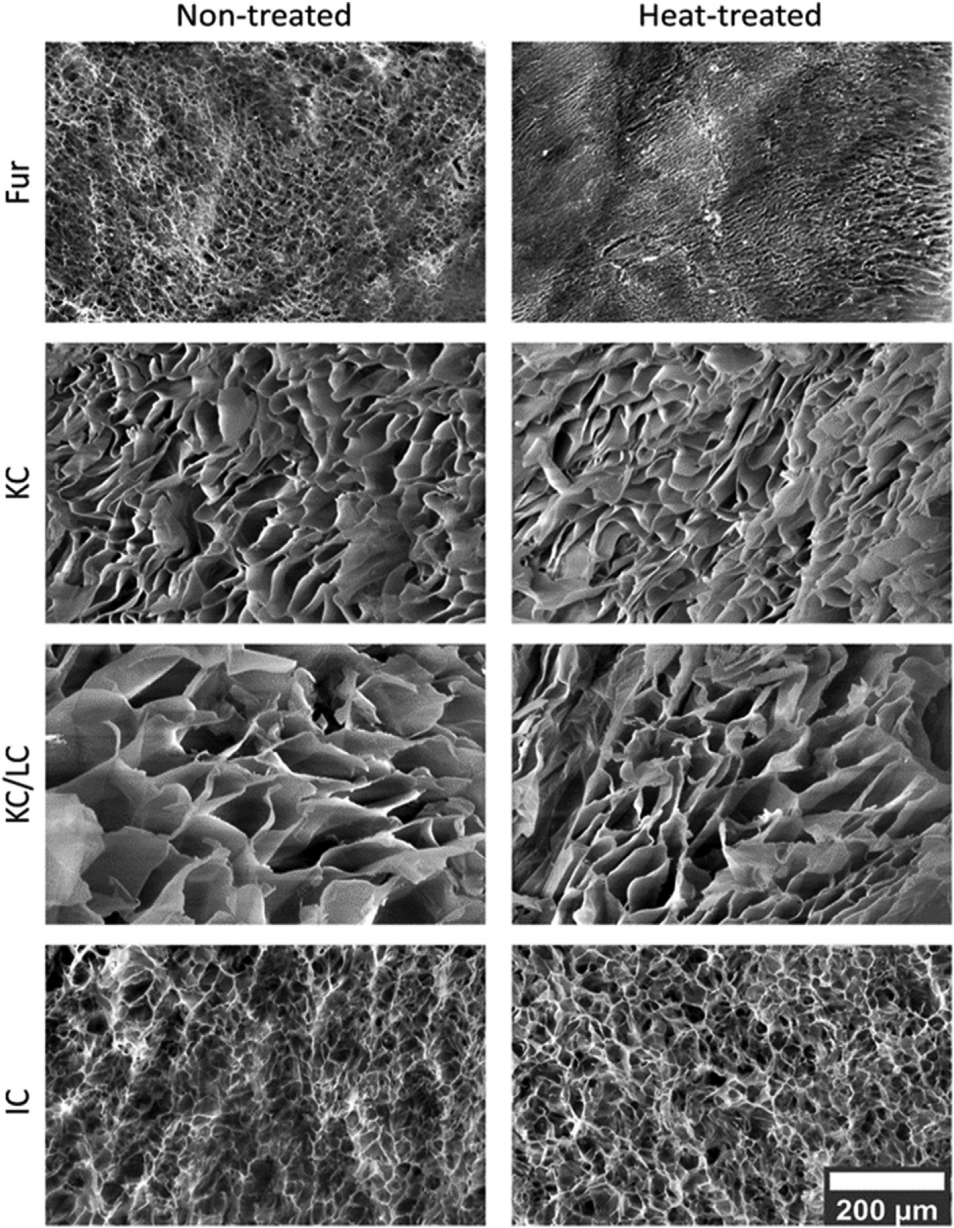
SEM images of 2.5% (w/w) gel network structures of non-treated and heat-treated (115 °C 15 min) carrageenans.

Changes in the morphology of the Fur and carrageenan gels before and after heat treatment were assessed using the SEM technique (Figure 8). It was observed that the gel skeleton structures of the hydrogel samples formed porous networks, Fur was similar to IC, and KC was similar to KC/LC. The first mentioned samples had a characteristic honeycomb structure, whereas KC and KC/LC exhibited long cross-linked tubular structures with rectangular pores. The effect of heat treatment was observed only for the Fur gel network, which lost the cross-linking, resulting in a decrease in gel hardness.

## 4 Conclusions

In this study, the influence of short-term heat treatment on the structure and rheological properties of commercial carrageenans was investigated. It was found that drying at 115 °C for 15 min had no influence on the structure of carrageenans except for Fur, where polysahharide degradation and galactan desulphation occurred. Heat decreased the molecular weight of carrageenans and the degradation was accelerated at higher temperatures. Fur had the lowest average molecular weight compared with the other carrageenans, as it had passed through previous severe repeated heat treatments in drum-drying during processing. It showed more sensitivity to heat as drying above 115 °C caused the loss of viscosity and gelling ability as the average molecular weight fell below the minimum value required for gelation. The viscosity and hardness of other studied carrageenan gels were not dependent on the drying temperature. All of the studied polysaccharide gels can be categorised as typical physically cross-linked network gels with thermo-reversible properties. Heat treatment decreases the storage modulus of carrageenan gels; also, the gelling and melting temperatures decrease with an increase in the heat treatment temperature, indicating that heat treatment impedes coil-helix transition. In order to improve the quality of carrageenans, the use of high temperatures in production must be avoided to prevent carrageenan degradation and functional property loss.

## Declarations

### Author contribution statement

Kairit Eha: Performed the experiments; Analyzed and interpreted the data; Wrote the paper.

Tõnis Pehk, Ivo Heinmaa, Aleksei Kaleda: Performed the experiments.

Katrin Laos: Conceived and designed the experiments; Analyzed and interpreted the data; Wrote the paper.

### Funding statement

This work was supported by the Estonian Ministry of Education (IUT19-27), Estonian Research Council (RESTA12 & RESTA13), and the 10.13039/501100008530European Regional Development Fund (RESTA12, RESTA13 & TK134).

### Data availability statement

Data will be made available on request.

### Declaration of interests statement

The authors declare no conflict of interest.

### Additional information

No additional information is available for this paper.
